# Heredity and vascular endothelium: focus on young age

**DOI:** 10.1186/1878-5085-5-S1-A84

**Published:** 2014-02-11

**Authors:** Maria E Evsevieva, Alexander J Krivoruchko, Tatiana A Smirnova, Maria V Rostovtseva, Oksana V Sergeeva, Ekaterina A Andreeva

**Affiliations:** 1 Stavropol State Medical University, Stavropol, Russia

## The actuality

In total mortality in all countries, almost 60% of deaths occur in cardiovascular disease (CVD) of atherosclerotic genesis [1]. It is proved that the roots of these diseases a thing of the young and even children years of life [2,3], so it is advisable to start prevention in youth, which requires development of prenosological criteria for the diagnosis of age-appropriate.

## Objective

To examine the state of the vascular endothelium in young people, taking into account the presence or absence of cases of early CVD in the immediate family.

## Material and methods

We investigated 40 students Stavropol State Medical University (16 boys and 24 girls) aged 19 to 22 who were divided into two groups: 1st group - with no family history (12 people) and the 2nd group - with heredity, burdened in the early CVD (28 people). The comparison group - 21 people from the hospital cardiac patients with severe forms of ischemic heart disease. Family history was regarded as burdened by the presence of CVD in the immediate family, like hypertension, stroke, and various forms of ischemic heart disease in men aged up to 55 years for women and 65 years. Blood was tested to estimate the number of desquamated or circulating endothelial cells by the method of J. Hladovec (1978) and a study of the profile of modifiable cardiovascular risk factors was fulfilled. The results were processed statistically using the software package BIOSTAT.

## Results

It turned out that the young people from the first group with a favorable inheritance of desquamated endothelial cells by an average of 2,6 ± 0,8, 2 median, mode 1, a maximum of 6 and a minimum of 1. These indicators are in the second group of students with a family history were equal respectively 5,8 ± 1,2, 4 median, mode 4, a maximum of 20 and a minimum of 2. That is, young people with a poor family history of the number of circulating endothelial cells was 2.2 times greater when compared with their peers who are not relatives of reported cases of early cardiovascular disease. These differences reached a statistically significant level (P ≥ 0,05). It is noteworthy that the number desquamated endothelial cells of patients with coronary artery disease is indicated by an average of 9,8 ± 1,5.

## Conclusion

Heredity, burdened by early CVD, at a young age is associated with changes in the vascular wall, vascular endothelial damage expressible with the phenomena of its increased desquamation. It is proved that endothelial dysfunction is the trigger mechanism of atherosclerosis and marker of preclinical stage flow [4,5]. These data are consistent with the results of multicenter studies that indicate a more prominent role unfavorable heredity as a risk factor (RF) of CVD is at a younger age compared to the more mature period of life when important prognostic role is played by modifiable risk factors [6]. The results confirm the importance of BH as screening parameters suitable for the formation of groups of cardiovascular risk among young people in order to carry out effective prevention programs in youth health centers [7] and the departments of medical prevention of student health centers that have recently begun to organize themselves quite extensively at various educational institutions the Russian Federation.
